# Autoimmune diseases and hypersensitivities improve the prognosis in ER-negative breast cancer

**DOI:** 10.1186/2193-1801-2-357

**Published:** 2013-07-30

**Authors:** Rickard Einefors, Ulrika Kogler, Carolina Ellberg, Håkan Olsson

**Affiliations:** Department of Oncology, Skåne University Hospital Lund, Barngatan 2 B, Lund, 221 85 Sweden; Department of Cancer Epidemiology, Skåne University Hospital Lund, Klinikgatan 22, Lund, 221 85 Sweden

**Keywords:** Breast cancer, Autoimmune diseases, Hypersensitivities, Immunostimulatory therapy, ER-negative, Premenopausal

## Abstract

**Introduction:**

Breast cancer (BC) is one of the leading causes of death among women worldwide. Immunostimulatory treatment has increasingly been used as adjuvant therapy in the last few years, in patients with melanoma and other cancer forms, often with an induction of autoimmunity as a consequence of a successful treatment. We aimed at investigating if coexisting autoimmune diseases (AD) or hypersensitivities (HS) similarly to the side effects of immunostimulatory treatment resulted in a better overall survival, compared to patients without these disorders.

**Material and methods:**

The patient material used was a consecutive clinical material consisting of 1705 patients diagnosed with BC between 1980 and 2010 in Sweden. The patients were stratified according to coexisting AD, HS or lack of both. Overall survival was calculated using Kaplan-Meier and the Cox proportional hazard model.

**Results:**

Our main finding was that BC patients with estrogen receptor (ER) negative tumors together with preexisting AD or HS had a statistically significant better overall survival (HR=0.53; 95% CI= 0.30-0.96) compared to patients without. Premenopausal BC patients with a coexistence of AD or HS had a better overall survival, but this was not statistically significant.

**Discussion:**

For patients with premenopausal or ER-negative BC, coexistence with AD or HS was associated with a better overall survival. Although these findings require validation, and the mechanisms responsible need to be found, they hint to possible new treatment strategies for BC, especially for those with ER-negative tumors and potentially for premenopausal patients.

**Electronic supplementary material:**

The online version of this article (doi:10.1186/2193-1801-2-357) contains supplementary material, which is available to authorized users.

## Introduction

Immunomodulatory antibodies have been used in phase III trials on metastatic melanoma to enhance anti-tumor immunity. One target protein studied in clinical trials for cancer treatment is Cytotoxic T-Lymphocyte Antigen 4 (CTLA-4). CTLA-4 is expressed on the surface of upregulated T-cells and has an inhibitory effect on T-cell activation. CTLA-4 plays an important role in the induction of tolerance to self-antigens (Sanderson et al. 2005; Attia et al. 2005). A blockade of CTLA-4 enhances anti-tumor immune actions and improves survival in metastatic malignant melanoma (Sanderson et al. 2005; Attia et al. 2005; Hodi et al. 2010; Simeone & Ascierto 2012). However, administration of this anti-CTLA-4 antibody is a double-edged sword. There is a strong correlation between the induction of tumor regression and grade 3/4 autoimmune toxicity. Another well-studied target protein of this kind is Programmed Death-1 (PD-1). PD-1 is expressed on activated T-cell and has an inhibitory effect on the effector phase of the T-cell response. A blockade of PD-1 has been shown to improve survival in various forms of stage 4 cancers. Adverse effects are mainly immune system related, but not as severe as those observed when using an anti-CTLA-4 antibody (Brahmer et al. 2012; Topalian et al. 2012).

Autoimmunity is the failure of an organism to recognize its own parts as self (Marrack et al. 2001; Atassi & Casali 2008). An epidemiological study in Denmark showed that the prevalence of 31 different AD in the population exceeded 5%. Diseases classified as AD in that study by Eaton *et al.* can be seen in Additional file 1: Table S1 (Eaton et al. 2007). Other conditions with a dysregulated immune system include asthma and allergies, which are both examples of HS. In both allergies and asthma, T-cell immune and inflammatory pathways are the main participants (Ahmad Al Obaidi et al. 2008; Cookson 2004; Kumar & Abbas 2007).

The hyperreactivity of the immune system in AD, allergies and asthma could possibly be beneficial for patients with cancer because of an increased tendency to attack the tumor cells. The mutated cancer cells have an altered protein expression and behavior, which enables the immune system to recognize them as malignant (Weinberg 2007). Hence, our hypothesis was that patients with AD or other HS such as asthma and allergies have a better overall survival rate compared to patients without AD or HS conditions. We decided to investigate this using BC as a model.

Cancer incidence among people with AD or HS has been studied previously, with conflicting results (Vojtechova & Martin 2009; Van Hemelrijck et al. 2010; Hwang et al. 2012; Hemminki et al. 2012; Turesson & Matteson 2012; Landgren et al. 2011). Cancer prognosis among the same patients has not been researched to the same extent and they focus mainly in specific diseases (Ji et al. 2011). The aim of this study was to investigate if BC patients with coexisting AD or HS have a better overall cancer survival compared to BC patients without these comorbidities.

## Material and methods

The study material was a cohort consisting of 1705 patients diagnosed with BC. Part of the patient material has been used in previous studies (Ellberg et al. 2012; Ellberg et al. 2010; Ellberg & Olsson 2011; Jernstrom et al. 1999). All patients were seen by the same physician at the Department of Oncology, Skåne University Hospital, Lund. The area of uptake is population-based and has been estimated to cover 300,000 patients in the southern parts of Sweden (overall population 1.5 million). The period of recruitment spans from Jan 1^st^ 1980 to Dec 31^st^ 2010. All subjects were interviewed through a standardized questionnaire filled out by the physician. This questionnaire contained information about date of birth, comorbidities, BC screening habits, parity, age of menarche, age of menopause, body mass index (BMI), ever-use of hormonal replacement therapy (HRT) and ever-use of oral contraceptive pills (OCP). Tumor stage, ER-status of the tumor and the date of diagnosis were gathered from pathological reports and patients’ charts. Follow-up was performed until Jan 1^st^ 2011 using the Swedish Civil Register as source. Informed consent was collected from all patients and the study was approved by an ethical board in Sweden (No 110–92).

The cohort was split in to three groups based on AD, HS and non-AD/HS. The criteria of inclusion for each group were report of having AD of any kind (y/n) (Additional file 1: Table S1), report of having type 1 HS, eczema or urticaria (y/n) and no report of having AD or HS, respectively. A fourth group (AD-HS) was constructed as well including both AD and HS. Throughout the entire study, the control group to an analyzed patient group was the corresponding counterpart of the cohort.

Any thyroid diseases of unspecified etiology found among recorded comorbidities were handled as follows. Patients with a reported diagnosis of hypothyroidism, hyperthyroidism, thyrotoxicosis or thyroiditis were included into AD. Patients with a reported diagnosis of goiters, levothyroxine use and thyroid surgery were excluded. Diabetes type I was included as an AD in the study. Diagnosed diabetes with the slightest uncertainty of type was classified as type II and hence excluded from the AD group.

ER-status was missing in 580 cases due to the fact that it was not performed in the clinic at the time of diagnosis with the exception of advanced BC. 341 cases were missing information about screening and out of these 315 were premenopausal. An assumption was made that none of these patients underwent screening, mainly due to young age.

We chose to study overall survival with age stratification. Other types of survival as breast cancer specific survival or relative survival were not studied. Two age cut-offs were used; the first at age 65 years old at time of BC diagnosis to reduce the influence on mortality due to comorbidities. The second cut-off was set at 50 years old at time of BC diagnosis to distinguish between pre- and postmenopausal. The median age of menopause in this material was 50 years.

### Statistics

All statistical analyses were performed using IBM SPSS 20.0. Overall survival for the AD, HS and AD-HS groups was estimated using Kaplan-Meier. For the AD-HS group the Kaplan-Meier analyses were also performed with stratification based on ER-status and menopausal status. The distribution was tested using the Log-rank test.

Hazard Ratios (HR) were calculated using cox proportional hazard. Both the AD and the HS groups were analyzed separately, adjusted for age at diagnosis of BC only, then adjusted for age at diagnosis of BC and TNM-stage and further with each age cut-off added. The AD-HS group was analyzed with the same adjustments initially and then adjustment for screening as well. The following variables were used: age at diagnosis of BC (linear), tumor size status (Tis, T1, T2, T3, or T4), and positive node status (N0, N1, N2, or N3), occurrence of distant metastasis (y/n) and screening (y/n).

HR for the AD-HS group was calculated in the subgroups of patients with ER-positive and ER-negative tumors and postmenopausal and premenopausal BC patients. Exclusion criteria included confirmed distant metastasis at diagnosis or carcinoma in situ. Exclusion criteria were set to reduce positive impact on survival in the AD-HS group and due to the low number of metastases. Adjustments were initially made for age at BC diagnosis, secondly age at BC diagnosis and T-stage and N-stage and finally adjustment for screening was added. The following variables were used: age at diagnosis of BC (linear), tumor size status (T1, T2, T3, or T4), and positive node status (N0, N1, N2, or N3) and screening (y/n).

All HR were estimated with 95% confidence intervals (CI). Two-tailed p-values were used for all analyses. A p-value of less than 0.05 was regarded as statistically significant.

## Results

### Patient material

Out of the 1705 BC patients in this study, 125 (7.3%) had an AD and 72 (4.2%) had HS (Table 1). At time of last follow-up 913 (53.5%) out of all patients were deceased. Median age at diagnosis of BC for the study population was 56.3 years old. Patients with an AD were somewhat older at diagnosis of BC (median 60.6 years) and patients with HS somewhat younger at BC diagnosis (median 53.3 years). A greater part (68.8%) of the BC patients with AD and a lesser part (54.2%) of the BC patients with HS had already entered menopause at time of their BC diagnosis. TNM-stage did not vary greatly between the groups. Similarly, neither BMI, age at menarche, age at menopause, HRT ever-use, nor parity showed any major differences between the groups. OCP ever-use was more common in the HS group (60%) than in the cohort as a whole (40%) (Table 2).

**Table 1:** **Summary of AD and HS diagnoses in the patient material**

a)		b)	
Autoimmune disease	No of cases	Hypersensitivity	No of cases
Reumatoid arthritis	28	Asthma	41
Thyreotoxicosis	16	Allergies	27
Hypothyroidism	15	Eczema	3
Psoriasis	12	Urticaria	1
Hyperthyroidism	8		
Ulcerous colitis	8	Total	72
Autoimmune gastritis	6		
Diabetes type 1	4		
Crohn’s disease	4		
Polymyalgia reumatica	4		
Thyroiditis	4		
Polyarthritis	3		
Mb Addison	2		
Multiple sclerosis	2		
Sjögrens’ disease	2		
Temporal arteritis	2		
Vitiligo	2		
Sacroidosis	1		
Systemic lupus erythematosus	1		
Wegeners’ granulomatosis	1		
Total	125

**Table 2:** **Patient and tumor characteristics in relation to coexistence of AD or HS**

		AD	HS	Non-hypersensitive	All
Number of cases		125	72	1508	1705
Age at diagnosis of BC	Median (range)	60.6 (31.5-80.4)	53.3 (25.4-82.5)	56.1 (23.6-89.5)	56.3 (23.6-89.5)
BMI	Median	24.6	24.1	24.2	24.2
Age at menarche	Median	13.0	13.0	13.5	13.5
Age at menopause	Median	50.0	50.0	50.0	50.0
Deceased	Yes	57 (46%)	30 (42%)	826 (55%)	913 (54%)
T-stage	In situ	4 (3%)	5 (7%)	71 (5%)	80 (5%)
	1	68 (54%)	36 (50%)	744 (49%)	848 (50%)
	2	40 (32%)	23 (32%)	539 (36%)	602 (35%)
	3	9 (7%)	8 (11%)	104 (7%)	121 (7%)
	4	0 (0%)	0 (0%)	7 (1%)	7 (0.4%)
	Unknown	4 (3%)	0 (0%)	43 (3%)	47 (3%)
N-stage	0	54 (43%)	39 (54%)	631 (42%)	724 (43%)
	1	44 (35%)	20 (28%)	514 (34%)	578 (34%)
	2	17 (14%)	8 (11%)	204 (14%)	229 (13%)
	3	5 (4%)	4 (6%)	91 (6%)	100 (6%)
	Unknown	5 (4%)	1 (1%)	68 (5%)	74 (4%)
M-stage	0	117 (94%)	71 (99%)	1349 (90%)	1537 (90%)
	1	1 (1%)	0 (0%)	24 (2%)	25 (2%)
	Unknown	7 (6%)	1 (1%)	135 (9%)	143 (8%)
ER Status	Negative	20 (16%)	17 (24%)	321 (21%)	358 (21%)
	Positive	70 (56%)	33 (46%)	664 (44%)	767 (45%)
	Unknown	35 (28%)	22 (31%)	523 (35%)	580 (34%)
Postmenopausal	No	32 (26%)	31 (43%)	490 (33%)	553 (32%)
	Yes	86 (69%)	39 (54%)	936 (62%)	1061 (62%)
	Unknown	7 (6%)	2 (3%)	82 (5%)	91 (5%)
Screening	No	78 (62%)	31 (43%)	934 (62%)	1043 (61%)
	Yes	29 (23%)	18 (25%)	274 (18%)	321 (19%)
	Unknown	18 (14%)	23 (32%)	300 (20%)	341 (20%)
OCP ever-use	Yes	50 (40%)	42 (58%)	625 (41%)	717 (42%)
HRT ever-use	Yes	31 (25%)	12 (17%)	277 (18%)	320 (19%)
Parity	0	28 (22%)	15 (21%)	250 (17%)	293 (17%)
	1	20 (16%)	15 (21%)	284 (19%)	319 (19%)
	2	56 (45%)	23 (32%)	579 (38%)	658 (39%)
	3	18 (14%)	10 (14%)	280 (19%)	308 (18%)
	4 +	3 (2%)	9 (13%)	115 (8%)	127 (7%)

### Univariate survival analysis

**Figure 1:**
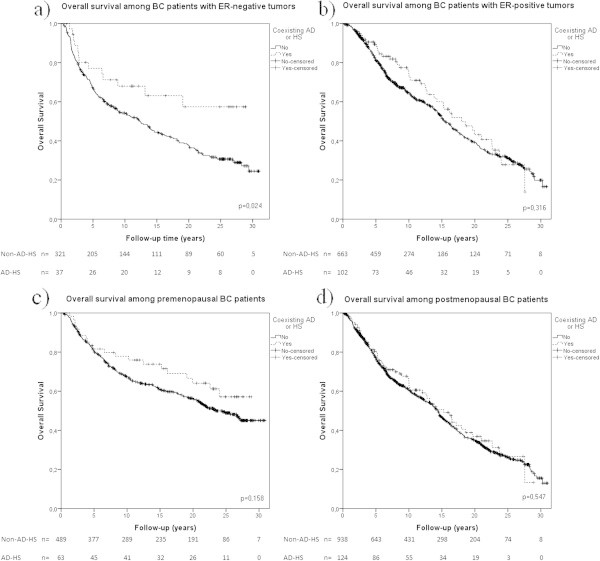
**Survival analysis in BC patients with coexistence of AD or HS stratified based on ER- and menopausal status.** Kaplan-Meier analysis on the coexistence of AD or HS stratified based on ER- and menopausal status in; **a)** patients with ER-negative tumors (dotted line) (N=36), **b)** patients with ER-positive tumors (dotted line) (N=96), **c)** premenopausal patients (dotted line) (N=56), and **d)** postmenopausal patients (dotted line) (N=117) compared to BC patients without (whole line). Log-rank tests were performed individually for each Kaplan-Meier analysis and are presented in each diagram respectively.

**Figure 2:**
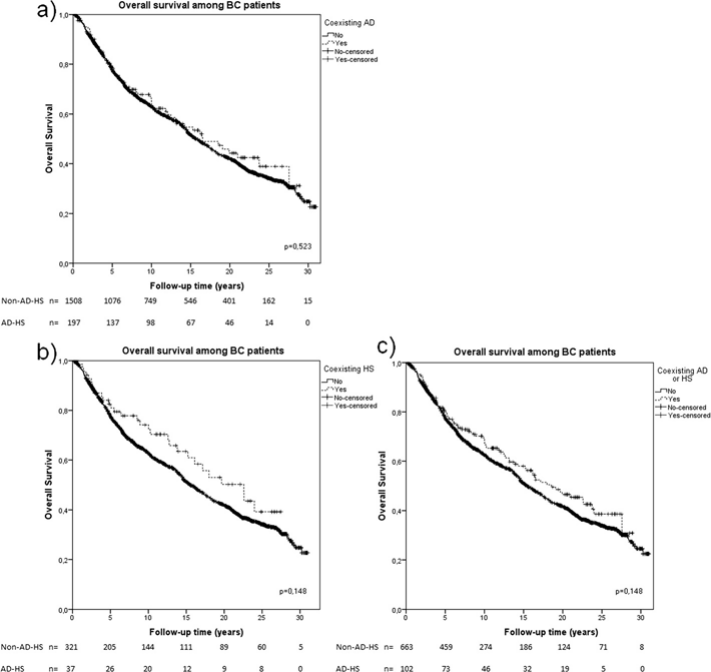
**Survival analysis in BC patients with coexistence of AD or HS.** Kaplan-Meier analysis on the coexistence of AD or HS in; **a)** patients with AD or HS (dotted line) (N=197), **b)** patients with AD (N=125) (dotted line), and **c)** patients with HS (dotted line) (N=72) compared to BC patients without (whole line). Log-rank tests were performed individually for each Kaplan-Meier analysis and are presented in each diagram respectively.

Overall survival was estimated using Kaplan-Meier. Stratified analyses were made according to ER-status and menopausal status using the AD-HS group. Patients with ER-negative tumors and AD-HS had a statistically significant better overall survival compared to patients without (Figure 1a). ER-positive cases with AD-HS had a non-statistically significant tendency towards better overall survival (Figure 1b). In premenopausal patients we found a non-statistically significant tendency towards better overall survival (Figure 1c). On the contrary, in postmenopausal patients coexistence of AD or HS did not affect the overall survival (Figure 1d). All three groups (AD-HS, AD, HS) analyzed separately had a tendency towards better overall survival, though none of these results were statistically significant (Figure 2).

### Multivariate survival analysis

Patients in the ER-negative subgroup with AD or HS had a statistically significant better overall survival compared to patients without, adjusted for age at BC diagnosis, TN-stage and screening (HR 0.53; CI 0.30-0.96) (Table 3a). The study did not show any difference in overall survival in the ER-positive subgroup between patients with AD or HS and patients without, adjusted for age at diagnosis of BC, TN-stage and screening (HR 0.98; CI 0.70-1.37) (Table 3a).

**Table 3:** **Cox proportional hazard stratified based on a) ER-status, and b) menopausal status relating BC patients with coexisting AD-HS to BC patients without**

a) Number of patients included in the analysis N=332 and N=695, respectively								
	Patients with ER-negative tumors				Patients with ER-positive tumors			
	HR	95% CI for HR		p-value	HR	95% CI for HR		p-value
		Lower	Upper			Lower	Upper	
AD-HS	**0.53**	**0.30**	**0.96**	**0.036**	0.98	0.70	1.37	0.909
T1	1.00	Ref	Ref		1.00	Ref	Ref	
T2	1.30	0.95	1.77	0.097	**1.60**	**1.26**	**2.03**	**<0.001**
T3	**2.62**	**1.69**	**4.08**	**<0.001**	**1.97**	**1.33**	**2.91**	**0.001**
T4	3.86	0.91	16.42	0.067	-	-	-	-
N0	1.00	Ref	Ref		1.00	Ref	Ref	
N1	**1.63**	**1.17**	**2.27**	**0.004**	1.28	1.00	1.64	0.054
N2	**2.29**	**1.51**	**3.49**	**<0.001**	1.38	0.98	1.93	0.064
N3	**2.72**	**1.54**	**4.81**	**0.001**	**2.61**	**1.68**	**4.06**	**<0.001**
Screening	0.59	0.27	1.26	0.174	0.75	0.51	1.11	0.149
**b) Number of patients included in the analysis N=499 and N=959, respectively**								
	**Premenopausal BC patients**				**Postmenopausal BC patients**			
	**HR**	**95% CI for HR**		**p-value**	**HR**	**95% CI for HR**		**p-value**
		**Lower**	**Upper**			**Lower**	**Upper**	
AD-HS	0.63	0.38	1.04	0.073	1.02	0.77	1.34	0.898
T1	1.00	Ref	Ref		1.00	Ref	Ref	
T2	1.22	0.91	1.65	0.190	**1.48**	**1.23**	**1.78**	**<0.001**
T3	**3.29**	**2.16**	**5.01**	**<0.001**	**1.73**	**1.25**	**2.39**	**0.001**
T4	-	-	-	-	1.68	0.53	5.31	0.377
N0	1.00	Ref	Ref		1.00	Ref	Ref	
N1	**1.54**	**1.11**	**2.13**	**0.009**	**1.58**	**1.28**	**1.95**	**<0.001**
N2	**2.72**	**1.85**	**4.00**	**<0.001**	**1.84**	**1.43**	**2.39**	**<0.001**
N3	**3.63**	**2.07**	**6.36**	**<0.001**	**2.95**	**2.15**	**4.05**	**<0.001**
Screening	0.66	0.29	1.50	0.319	**0.76**	**0.59**	**0.97**	**0.030**

Both models are adjusted for age at BC diagnosis, T- and N-status of the tumor, and screening simultaneously.

T1 and N0 were reference values for respective category.

In premenopausal BC patients, AD or HS was associated with a tendency towards better overall survival adjusted for at age at BC diagnosis, TN-stage and screening (HR 0.63; CI 0.38-1.04) (Table 3b). Postmenopausal patients with AD or HS had no tendency towards better or worse overall survival adjusted for age at BC diagnosis, TN-stage and screening (HR 1.02; CI 0.77-1.34) (Table 3b).

Among all patients diagnosed with BC, those with coexisting AD or HS had a non-statistically significant tendency towards better overall survival adjusted for age at diagnosis of BC (HR 0.88; CI 0.71-1.10). Adding adjustment for TNM-stage as well, a better overall survival effect remained but not statistically significant (HR 0.91; CI 0.72-1.14). Additional adjustment for screening showed a non-statistically significant tendency towards better overall survival (HR 0.95; CI 0.74-1.23). Including only patients younger than 50 years old at BC diagnosis in the analysis the AD-HS group had a tendency towards a better overall survival (HR 0.74; CI 0.48-1.16) (Table 4a).

**Table 4:** **Cox proportional hazard relating coexistence of a) AD-HS, b) AD, and c) HS to overall survival compared to BC patients without these comorbidities**

a) BC patients with coexisting AD or HS										
	All patients. N=1508, N=1363 and N=1363.									
		HR	95% CI for HR		p-value					
AD-HS, adjusted for:	N		Lower	Upper						
Age at diagnosis	194	0.88	0.71	1.10	0.25					
Age at diagnosis, TNM-stage	185	0.91	0.72	1.14	0.409					
Age at diagnosis, TNM-stage, Screening	185	0.94	0.75	1.18	0.579					
	Patients younger than 65 years at diagnosis of BC. N=1021					Patients younger than 50 years at diagnosis of BC. N=473				
	N	HR	95% CI for HR		p-value	N	HR	95% CI for HR		p-value
AD-HS, adjusted for:			Lower	Upper				Lower	Upper	
Age at diagnosis, TNM-stage	130	0.94	0.75	1.18	0.579	56	0.74	0.48	1.16	0.186
**b) BC patients with coexisting AD**										
	All patients. N=1580 and N=1433									
	N	HR	95% CI for HR		p-value					
AD adjusted for:			Lower	Upper						
Age at diagnosis	122	0.90	0.48	1.16	0.460					
Age at diagnosis, TNM-stage	115	0.90	0.69	1.20	0.480					
	Patients younger than 65 years at diagnosis of BC. N=1071					Patients younger than 50 years at diagnosis of BC. N=500				
	N	HR	95% CI for HR		p-value	N	HR	95% CI for HR		p-value
AD adjusted for:			Lower	Upper				Lower	Upper	
Age at diagnosis, TNM-stage	80	0.95	0.66	1.36	0.773	29	0.81	0.45	1.45	0.472
**c) BC patients with coexisting HS**										
	All patients included. N= 1632 and N=1363									
	N	HR	95% CI for HR		p-value					
HS adjusted for:			Lower	Upper						
Age at diagnosis	72	0.85	0.60	1.23	0.388					
Age at diagnosis, TNM-stage	70	0.93	0.64	1.34	0.691					
	Patients younger than 65 years at diagnosis of BC. N=1101					Patients younger than 50 years at diagnosis of BC. N=502				
	N	HR	95% CI for HR		p-value	N	HR	95% CI for HR		p-value
HS adjusted for:			Lower	Upper				Lower	Upper	
Age at diagnosis, TNM-stage	50	0.86	0.54	1.37	0.524	27	0.69	0.36	1.33	0.269

The HR shown is for a) AD-HS combined, b) AD, or c) HS adjusted for the variables in the left column.

All analyses were performed using all patients, with an age cutoff at less than 65 years old, and an age cutoff at less than 50 years old for each group respectively.

Both the AD and HS groups had a tendency towards better overall survival when they were analyzed separately (Table 4b, 4c). None of these results were statistically significant.

## Discussion

Our aim was to investigate if AD or HS constitute protective factors for overall survival among BC patients. This study showed a statistically significant overall survival benefit for patients diagnosed with an ER-negative BC if they had a concurrent diagnosis of an AD or a HS. Premenopausal BC patients with AD or HS had an overall survival advantage compared to those without these comorbidities.

This raises the question of the mechanism behind the better overall survival for patients with co-existing AD or HS. As mentioned in the introduction, successful immunostimulatory therapy is often associated with the development of an AD. Since the mutated cancer cells have developed host immune evasion strategies, an overactive immune system that reacts to self-antigens may discover and target cancer cells more readily. From our study, these benefits were more evident in young patients and in those ER-negative BC, but were less clear for other patients.

In most analyses, the difference between the groups was weakened when adjusted for tumor size, lymph node involvement and distant metastases. A possible explanation for this might be that patients with AD or HS had less advanced tumors at time of diagnosis and/or an effect of exclusion of patients with missing information. It might also be that coexistence of AD or HS retards or inhibits tumor progression. Another possible reason is that patients with AD or HS have closer and more regular contact with medical health care and therefore the tumors are found at an earlier stage. Lower mortality observed among patients with AD or HS may also represent the effect of a generalized increase in health awareness.

ER-negative BC patients with AD or HS still exhibited significant overall survival differences even after adjusting for tumor size and lymph node involvement. Furthermore, adjustment for BC screening was made, since BC found through screening has a better prognosis due to earlier detection (Nystrom et al. 2002). Among patients with ER-negative tumors, screening detection did not weaken the result. This indicates that TN-stage and screening detection probably do not influence the longer overall survival for patients with ER-negative tumors and coexisting HS or AD.

Why AD and HS is associated with a better overall survival specifically among the ER-negative BC patients is not known. Compared to ER-positive tumors, the ER-negative are associated with a poorer prognosis, fewer treatment strategies and in generally, a worse tumor grade and hence a more aggressive biological behavior (Putti et al. 2005). Considering the results from our study, there seems to be a protective effect from AD or HS which possibly contributes to decrease the tumor progression. In a recent study by Calabro *et al.*, it is indicated that lymphocyte infiltrate (LI) is associated with survival, but it has opposite effects in ER-positive compared to ER-negative BC patients. A high LI of ER-positive BC patients was associated with a worse prognosis. However, a high LI in ER-negative BC patients was associated with a better overall survival for these patients. LI occurs as a reaction of the organism to the growing tumor mass. Further, Calabro *et al.*, suggest, that the results might reflect intrinsic differences in the biology of the breast-tumor subtypes and also a difference in tumor immune surveillance. ER-status seems to be important for this differentiation (Calabro et al. 2009). This finding points in a similar direction as our result for the ER-negative BC patients, in which they both suggest a specific molecular mechanism through which the immune system effectively inhibits growth of ER-negative tumors. Our suggestion is that LI is higher in AD or HS patients, compared with patients without these conditions. Given that our theory is true, another conceivable explanation for our result could therefore be that an ER-negative BC with a high LI, perhaps caused by an AD or HS, after given therapy more efficiently attacks the tumor cells, than an ER-negative tumor with a low LI. For the ER-negative tumors, where no satisfactory treatment is available, the LI serves as an important supplement defense line for the body.

### Possible bias

The patients reported occurrence of AD or HS to the treating doctor during the interview at time of BC diagnosis. There might be some BC patients that omitted to report or forgot anything of importance for our study. Additionally, it is impossible to know whether there was a selection bias in the group of patients that perhaps did not report. In some instances, other data, such as ER-status, occurrence of metastases, lymph node involvement etc. was missing due to clinical practice at the time of diagnosis.

ER-status was missing in 34% of all patients (Table 2). Most missing data was from the patients diagnosed earlier than the mid-nineties, which was before determination of ER-status was a part of regular clinical practice. Because the grade of selection is low, this bias factor should not be too worrying for our result. The patients diagnosed at the time when ER-status was brought in to clinical practice can show a selection bias though. At the start phase, it is possible that ER-status was taken only on some specially prioritized patients, for example those with large and aggressively growing tumors or those with hereditary BC.

The definition of an AD is somewhat fluctuating which is an issue for discussion. We mainly utilized the Danish definition from an extensive epidemiological study in 2007 (Additional file 1: Table S1). This choice of definition could have meant that some of the diseases are misclassified. But it should be remembered that this is an exploratory study, which means that our results could be due to chance and they need to be validated in an independent material.

Diabetes and diseases of the thyroid that did not meet certain criteria as described in *Materials and Methods*, were excluded from AD. Therefore, diabetes and thyroid issues with autoimmune etiology might have been incorrectly included and excluded. However, this would only have weakened our result, which might mean that a stronger effect exists in reality.

Patients in the southern health care region in Sweden with BC that need radio- and/or chemotherapy are referred to the Department of Oncology, Skåne University Hospital, Lund. However, the region has been changing slightly during the 1980s and 1990s, but this should not influence our results significantly. Patients who were not treated with radio- and/or chemotherapy or who were too old and weak to receive this treatment and those whose cancer was too advanced were not remitted to the Department of Oncology, Skåne University Hospital, Lund. This means, that the median age is slightly younger in our material than that of BC patients in general. In the end, the low median age entails in a result that is not completely generalizable on all BC patients. In our study, data collection and treatment of patients, stretches over a long time span (1980–2010). During this period of time, criteria for diagnosis and available treatments have varied, chiefly for BC, but also for AD, which is another reason why these results needs to be validated in an independent material.

## Conclusion

Patients with ER-negative BC had a statistically significant better overall survival when they had a history of AD or a HS. The overall survival was better among premenopausal patients with AD or HS compared to patients without AD or HS. These findings need to be validated in an independent material. If an over-activated immune system that accompanies a coexisting AD or HS gives a longer survival for BC patients, an immune activation is a possible future target for treatment, especially for patients with ER negative tumors and for young patients.

### Ethical standards

This study has been approved by an ethical board and complies with the current laws of Sweden.

## Electronic supplementary material

Additional file 1: Table S1: Diseases generally considered as autoimmune: thyrotoxicosis, Autoimmune thyroiditis, Insulin dependent diabetes, Primary adrenocortical insufficiency , Celiac disease, Pernicious anemia, Autoimmune hemolytic anemia, Idiopathic thrombocytopenic purpura, Multiple sclerosis, Guillain Barre syndrome, Iridocyclitis, Wegener’s granulomatosis, Crohn’s disease, Ulcerative colitis, Primary biliary cirrhosis, Chronic hepatitis, Interstitial cystitis, Endometriosis, Pemphigoid, Pemphigus, Psoriasis vulgaris, Alopecia areata, Vitiligo, Seropositive rheumatoid arthritis, Dermatopolymyositis, Myositis, Polymyalgia rheumatica, Myasthenia gravis, Systemic sclerosis, Systemic lupus erythematosis, Sjogren’s syndrome. (BMP 1 MB)

## Abbreviations

AD: Autoimmune diseases
BC: Breast cancer
BMI: Body mass index
CI: Confidence interval
CTLA-4: Cytotoxic T-Lymphocyte Antigen 4
ER: Estrogen receptor
HR: Hazard ratio
HRT: Hormonal replacement therapy
HS: Hypersensitivities
LI: Lymphocyte infiltration
OCP: Oral contraceptive pills.
