# Supplementary figures and images for: Autoimmune diseases and hypersensitivities improve the prognosis in ER-negative breast cancer
Source: Springerplus. 2013 Jul 30;2:357. doi: 10.1186/2193-1801-2-357 (PMC3755812; doi:10.1186/2193-1801-2-357)

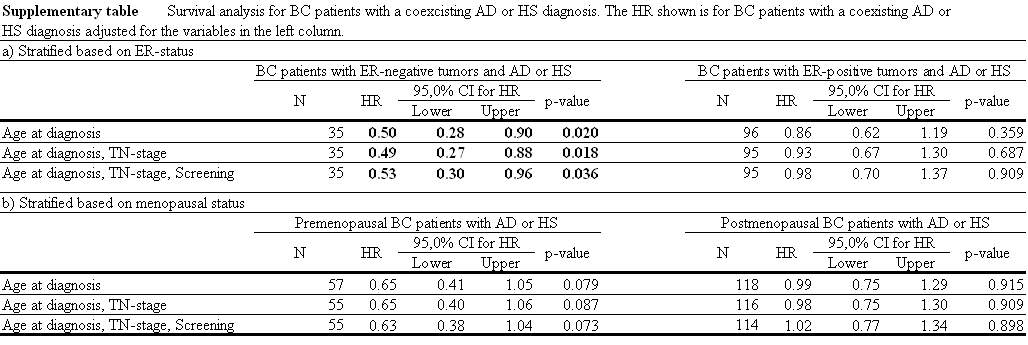

Supplement: Supplementary file 1 — Additional file 1: Table S1: Diseases generally considered as autoimmune: thyrotoxicosis, Autoimmune thyroiditis, Insulin dependent diabetes, Primary adrenocortical insufficiency , Celiac disease, Pernicious anemia, Autoimmune hemolytic anemia, Idiopathic thrombocytopenic purpura, Multiple sclerosis, Guillain Barre syndrome, Iridocyclitis, Wegener’s granulomatosis, Crohn’s disease, Ulcerative colitis, Primary biliary cirrhosis, Chronic hepatitis, Interstitial cystitis, Endometriosis, Pemphigoid, Pemphigus, Psoriasis vulgaris, Alopecia areata, Vitiligo, Seropositive rheumatoid arthritis, Dermatopolymyositis, Myositis, Polymyalgia rheumatica, Myasthenia gravis, Systemic sclerosis, Systemic lupus erythematosis, Sjogren’s syndrome. (BMP 1 MB) [file 40064_2013_444_MOESM1_ESM.bmp]
